# Application of a Web-Enabled Leg Training System for the Objective Monitoring and Quantitative Analysis of Exercise-Induced Fatigue

**DOI:** 10.2196/resprot.4985

**Published:** 2016-08-22

**Authors:** Vadim N Dedov, Irina V Dedova

**Affiliations:** ^1^MedExercise ProjectResearch and DevelopmentMDXD Pty LtdSydneyAustralia; ^2^Department of AnatomySchool of Medical SciencesUniversity of New South WalesSydneyAustralia

**Keywords:** exercise intervention, cardiac rehabilitation, training equipment, online monitoring, exercise dose, muscle fatigue, heart rate, leg work output, fatigability

## Abstract

**Background:**

Sustained cardiac rehabilitation is the key intervention in the prevention and treatment of many human diseases. However, implementation of exercise programs can be challenging because of early fatigability in patients with chronic diseases, overweight individuals, and aged people. Current methods of fatigability assessment are based on subjective self-reporting such as rating of perceived exertion or require specialized laboratory conditions and sophisticated equipment. A practical approach allowing objective measurement of exercise-induced fatigue would be useful for the optimization of sustained delivery of cardiac rehabilitation to improve patient outcomes.

**Objectives:**

The objective of this study is to develop and validate an innovative approach, allowing for the objective assessment of exercise-induced fatigue using the Web-enabled leg rehabilitation system.

**Methods:**

MedExercise training devices were equipped with wireless temperature sensors in order to monitor their usage by temperature rise in the resistance unit (Δ*t*°). Since Δ*t*° correlated with the intensity and duration of exercise, this parameter was used to characterize participants’ leg work output (LWO). Personal smart devices such as laptop computers with wireless gateways and relevant software were used for monitoring of self-control training. Connection of smart devices to the Internet and cloud-based software allowed remote monitoring of LWO in participants training at home. Heart rates (HRs) were measured by fingertip pulse oximeters simultaneously with Δ*t*° in 7 healthy volunteers.

**Results:**

Exercise-induced fatigue manifested as the decline of LWO and/or rising HR, which could be observed in real-time. Conversely, training at the steady-state LWO and HR for the entire duration of exercise bout was considered as fatigue-free. The amounts of recommended daily physical activity were expressed as the individual Δ*t*° values reached during 30-minute fatigue-free exercise of moderate intensity resulting in a mean of 8.1°C (SD 1.5°C, N=7). These Δ*t*° values were applied as the thresholds for sending automatic notifications upon taking the personalized LWO doses by self-control training at home. While the mean time of taking LWO doses was 30.3 (SD 4.1) minutes (n=25), analysis of times required to reach the same Δ*t*° by the same participant revealed that longer durations were due to fatigability, manifesting as reduced LWO at the later stages of training bouts. Typically, exercising in the afternoons associated with no fatigue, although longer durations of evening sessions suggested a diurnal fatigability pattern.

**Conclusions:**

This pilot study demonstrated the feasibility of objective monitoring of fatigue development in real-time and online as well as retrospective fatigability quantification by the duration of training bouts to reach the same exercise dose. This simple method of leg training at home accompanied by routine fatigue monitoring might be useful for the optimization of exercise interventions in primary care and special populations.

## Introduction

Regular cardiac (aerobic/endurance) training is essential in the maintenance of health and considered as a key intervention in prevention, treatment, and rehabilitation of many human diseases [1]. However, cardiac rehabilitation (CR) remains vastly underutilized due to significant barriers such as the high costs of services and low exercise capacity of patients [2]. The most popular CR regimen is moderate-intensity continuous training (MICT). The consensus among experts is that MICT bouts should be performed at least 5 days per week for 30 minutes per day or 150 minutes per week [3,4]. These guidelines are similar for the general, clinical, and special populations.

It has been proposed that sustainable CR should be personalized because of variability in the individual fitness levels and physiological responsiveness to physical activity [5]. A conventional approach to personalization of CR is expressing the exercise intensity as a percentage of the maximal oxygen uptake (VO_2max_) or heart rate (HR_max_) [6]. It is generally accepted that the intensity of MICT in clinical and general populations should be within 50% to 70% of the VO_2max_ or HR_max_[7]. However, the typical duration of conventional exercise tests does not exceed 10 minutes so that they may not fully reflect the changes in physiological responses at the later stages of the recommended 30-minute or longer MICT sessions [8]. Development of exercise-induced fatigue is expected to manifest as the slowing down of exercise intensity at the later stage of training bouts, particularly in untrained people [9].

Fatigue is not only the normal physiological response to physical activity but also the most common symptom of human disease and sign of ageing [10,11]. Further, even a relatively short immobilization results in markedly decreased levels of fatigue resistance [9]. There is also chronic fatigue syndrome, which is characterized by persistent fatigue and treated by physical exercise [12]. Therefore, CR programs prescribed to patients with chronic disease, overweight individuals, and aged persons should consider their higher fatigability than the general population [13,14]. However, despite the potential diagnostic and predictive value of fatigue measurements [15], clinically valid methods suitable for the assessment of fatigability are still lacking [16].

The existing methods used in clinical practice include the subjective rating of perceived exertion (RPE) [17,18], which is based on self-reporting and hence might be biased, particularly in obese participants [19]. Objective measurements of muscle fatigue require laboratory conditions and sophisticated equipment [20]. For example, a typical laboratory protocol involves electromyographic recordings from the participant using incremental cycle ergometry or treadmill running [21]. Modern technology facilitated the development of wearable measuring systems such as a fatigue monitoring system and body sensor network [22,23], although they have yet to be implemented in practice.

Therefore, the current methods of fatigue assessment are either subjective or based on complex technologies. In this study we report research protocols allowing for the monitoring and quantification of fatigue during routine exercise training with an innovative leg rehabilitation system [24,25]. Remote fitness testing, personalization of MICT doses, and quantification of fatigability demonstrated in this study might be useful for the optimization of CR [26].

## Methods

### Equipment and Data Collection

The MedExercise ST01 leg training system (MDXD Pty Ltd, Sydney, NSW, Australia) was used in this study. This portable system was developed for exercise rehabilitation on site and consists of a variable resistance unit with two pedals, means for attachment to the furniture, and a measurement module [24,25]. The resistance unit of the system is equipped with wireless temperature sensors (Monnit, Midvale, UT) positioned in two different locations allowing for the measurement of temperature at high and low sensitivity modes. Temperature data was transmitted in 1 minute intervals to the user’s electronic device such as personal computers via MonnitLink Universal Serial Bus (USB) gateways and processed with stand-alone or cloud-based iMonnit software.

Before each training session, the temperature values were set to zero. The exercise-induced rise of temperature in the resistance unit (Δ*t*°) was used for the measurement of a total leg work output (LWO). Since Δ*t*° depends on the resistance to foot motion, cadence of leg movements, and training duration, LWO reflects the energy expenditure of the user, similar to the calorie counting in cycle ergometers [24,25]. The automatic notification feature of iMonnit software allowed for the setting of email alerts upon reaching specified Δ*t*°, reflecting the total LWO of the participant during a training bout [26].

Heart rates (HRs), expressed as a number of beats per minute (BPM), were monitored using fingertip pulse oximeters CMS-50E and the software supplied with it (Contec, Shanghai, China). HR was graded as the percentage to HR_max_ using the following conventional formula:

HR_max_=220-age

The RPE was assessed using the Borg’s scale 6 to 20 [27] and ranked as light (<12), moderate (12-14) or strenuous to continue (>14). Data was processed and analyzed using Excel worksheets software (Microsoft, Redmond, WA).

### Study Design

It was hypothesized that the simultaneous monitoring of exercise intensity and participant’s performance by HR and LWO, respectively, might detect the development of fatigue during exercise training. In order to test this hypothesis, two experimental settings were used. First, supervised training bouts at different intensities and durations were performed at the high and low sensitivities of LWO measurement. Then, the system was used at low-sensitivity for self-controlled training at home with automated monitoring of compliance. The duration of training and number of participants corresponded to the aim of the study; protocol development for the quantitative measurement of exercise-induced fatigue and fatigability.

### Participants

The inclusion criteria were (1) absence of known medical contraindications to regular exercise training; (2) capacity to install the training equipment and use it at home; and (3) connection to the Internet and ability to manage electronic data. The reasons for exclusion included (1) inability to provide informed consent; (2) not adhering to a prescribed training regimen; and (3) failure to use technology reliably. Overall, 7 eligible volunteers (25 to 52 years old) participated from 2013 to 2015.

## Results

### High-Sensitivity Recording

**Figure 1:**
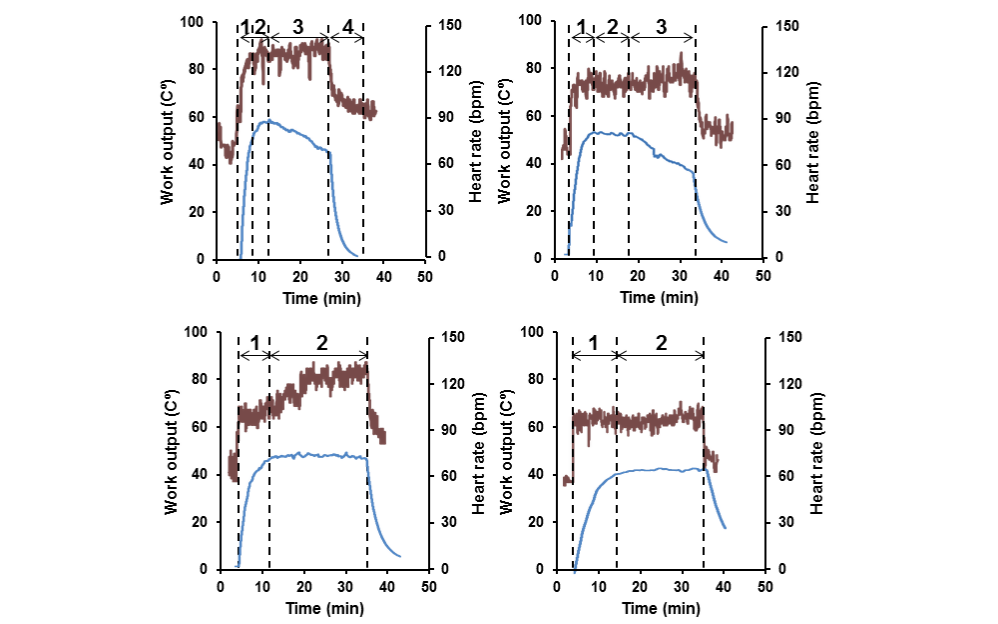
High-sensitivity Δ*t*° measurements showing the simultaneous recording of participant LWO (left scale, blue curve) and HR (right scale, brown curve) during vigorous >130 (SD 10) BPM (top left), vigorous-moderate 120 (SD 10) BPM (top right), variable 100-130 (SD 10) BPM (bottom left), and moderate 100 (SD 10) BPM (bottom right) levels of exercise intensity. Intervals 1-4 on top of arrows indicate Δ*t*° phases during exercise bouts of various intensities.

The monitoring of participant’s performance during exercise bouts of various intensities using Δ*t*° to characterize LWO is exemplified in Figure 1. Vigorous training, as indicated by HRs over 130 BPM or greater than 70% HR_max_[28], is illustrated in the top left panel of Figure 1. The RPE rating was considered more than 14 throughout the entire exercise bout. The Δ*t*° rose to approximately 57°C (interval 1), plateaued for a few minutes (interval 2), and then progressively declined (interval 3) despite a steady state HR. Upon cessation of training, both the participant’s HR and Δ*t*° in the training device dropped (interval 4). This experiment demonstrates that reduction of LWO (interval 3) started after around 7 minutes of training despite the participant maintaining a high intensity level of exercise as indicated by a HR over 130 BPM.

The LWO kinetics at a HR of 120 (SD 10) BPM or around 70% HR_max_, moderate to vigorous intensity levels of exercise for this participant, is shown in the top right panel of Figure 1. The corresponding RPE score was considered at first as 12 to 14 and then over 14. Compared to the vigorous intensity of training (Figure 1, top left), a lesser intensity of exercise caused a slower rise and lower maximum Δ*t*° of around 51°C. The plateau level of LWO lasted about 9 minutes before a gradual reduction so that the duration of steady-state performance was about 15 minutes, the sum of intervals 1 and 2.

The top panels in Figure 1 demonstrate an inverse correlation between training intensity and the participant’s ability to maintain LWO at the steady-state level, for example for 7 and 15 minutes, respectively. An inverse correlation between these parameters suggests that participants’ muscle fatigue manifest as a reduction of LWO despite the same intensity of training.

The bottom left panel of Figure 1 shows recordings from the experiment, which intended to reproduce the recommended amount of daily exercise achieved by 30-minute MICT [4]. The corresponding initial exercise intensity was 100 (SD 10) BPM or 55% to 70% HR_max_, indicating a moderate intensity of exercise. The steady-state level of LWO with the RPE of 12 to 14 was maintained for the entire 30 minutes of the training bout. Nevertheless, the HR was moderate only for about 10 minutes and then gradually rose to the maximum of approximately 130 BPM or greater than 70% HR_max_. This suggests that maintaining a steady-state LWO at this level requires progressive increasing of exercise intensity from the moderate to vigorous level as indicated by a rising HR from 100 to 130 BPM.

In contrast, the bottom right panel of Figure 1 exemplifies a 30-minute MICT session at a HR of 100 (SD 10) BPM, where neither muscle fatigue (reduction of LWO) nor the increase in exercise intensity (rising HR) was manifested. The RPE score was 12 to 14. Therefore, this training session might be considered as a fatigue-free MICT bout matching the recommended “dose” of daily exercise for this participant. It might be suggested that repeating this amount of training at least 5 days per week would match the recommended weekly volumes of exercise [4].

**Figure 2:**
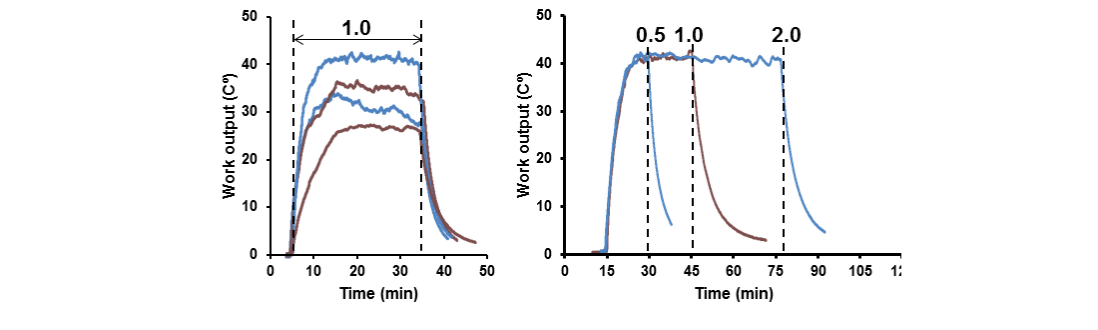
High-sensitivity Δ*t*° measurements showing variability of Δ*t*° at moderate exercise intensity of 100 (SD 10) BPM (left) and Δ*t*° recordings of the same participant training at the HR of 100 (SD 10) BPM for 15, 30, and 60 minutes on different days (right). Vertical dashed lines and numbers indicate amount of daily training as a proportion to the recommended 1.0 dose of MICT [4].

The variability of LWO patterns between the participants during 30-minute MICT at a HR of 100 (SD 10) BPM is demonstrated in Figure 2 (left panel). The corresponding RPE scores were 12 to 14. These recordings exemplify individual differences in the LWO profiles demonstrated by the variability of plateaus levels, where Δ*t*° varied from 24°C to 42°C, with a mean of 34.2°C (SD 5.7°C, N=7).

The LWO recordings of the participant, who performed MICT bouts of various durations on different days, are shown in Figure 2 (right panel). Since the Δ*t*° plateau levels were reproducible, the volumes of MICT were proportional to the duration of training. In this example, MICT for 15, 30, and 60 minutes could be considered as corresponding to 0.5, 1.0, and 2.0 daily exercise doses for this participant [4]. The MICT volume equal to or less than 1.0 indicates “taking” a recommended daily exercise dose, whereas a value 0.5 is a half of the dose, and a value 2.0 means that two daily MICT doses were “taken” during a single exercise bout.

### Low-Sensitivity Recording

**Figure 3:**
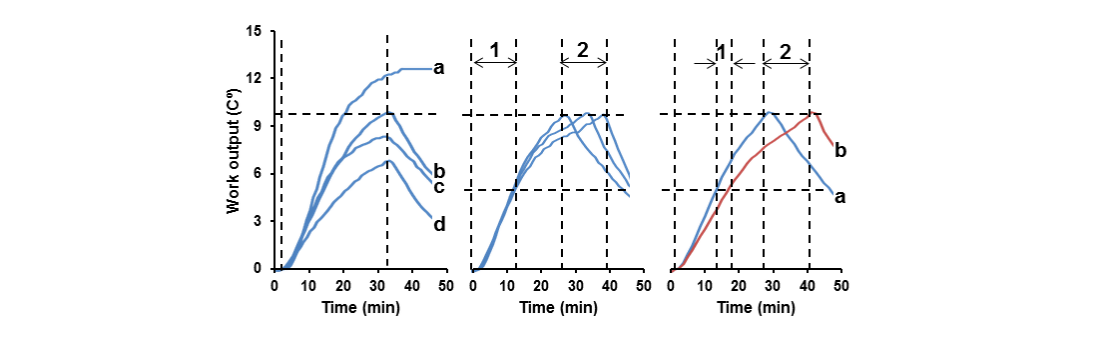
Low-sensitivity recordings of changes in Δ*t*° at a HR of 120 (SD 10) BPM (curve a) and 100 (SD 10) BPM (curves b-d) (left panel). Low-sensitivity recordings of different durations to reach Δ*t*° dose of 10°C on different days by the same participant (middle) and two consecutive bouts to reach a Δ*t*° dose of 10°C by the same participant consecutively using two training devices (right). Vertical dashed lines represent durations needed to reach a half (arrows 1) and the full LWO dose of 10°C (arrows 2) delineated by horizontal dashed lines.

**Figure 4:**
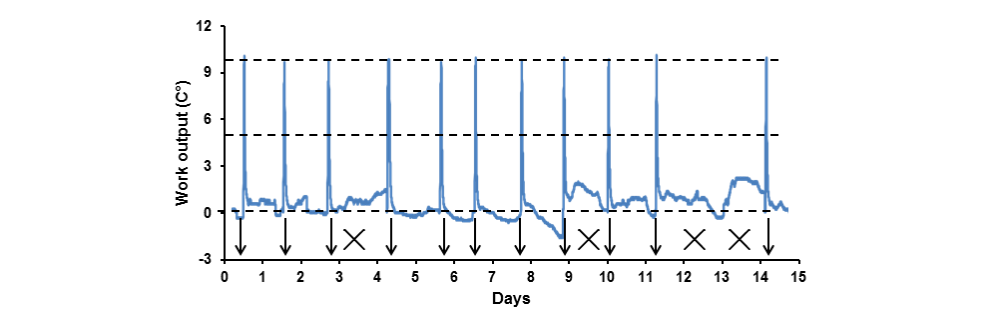
Low-sensitivity recordings of a continuous Δt° recording from the training device installed in a participant’s home during 15 days. Horizontal dashed lines indicate the levels of "zero", half, and full LWO dose of 10°C. Arrows indicate days and times of the day when the automated notifications were sent. Crosses mark days without training with the device.

While high-sensitivity measurements allow real-time visualization of fatigue development, quantification of MICT doses is complicated due to variability of LWO plateau levels, as exemplified in Figure 2 (left panel). It is also evident that the HR reflects the intensity of training, whereas the right panel in Figure 2 suggests that Δ*t*° could be used for selecting daily exercise doses. Conversely, we used a low-sensitivity mode to express the daily MICT doses in single-item numbers, as shown in Figures 3 and 4.

Low-sensitivity recordings of the participant, whose high-sensitivity recording were presented in Figure 1 (top right) is shown in Figure 3 (left panel, curve a). In both cases, the participant was training vigorously at a HR of 120 (SD 10) BPM, but low-sensitivity did not allow Δ*t*° to plateau within at least 30 minutes of training. This suggests that during less intensive exercise, (eg, MICT), rising Δ*t*° should be linear without reaching the saturation level within 30 minutes. Therefore, Δ*t*° achieved by MICT in 30 minutes would reflect the daily dose of exercise [4]. Curves b-d (Figure 3, left) exemplify low-sensitivity LWO recordings of participants training at HRs of 100 (SD 10) BPM for 30 minutes to reach the recommended amounts of daily MICT. The associated RPE scores were 12 to 14. This shows that Δ*t*° values varied from 7.1°C to 10.0°C and could be considered the personalized doses of recommended daily MICT for these participants expressed as single numbers. The mean Δ*t*° is 8.1°C (SD 1.5°C, N=7).

Next, we tested the reproducibility of “taking” the personalized LWO doses expressed in Δ*t*°, and the recordings of 3 MICT sessions at a HR of 100 (SD 10) BPM performed by the same participant on different days to reach a personalized Δ*t*° dose of 10°C are shown in Figure 3 (middle). While the dose was reached on all occasions, their duration varied between 26 and 37 minutes (arrow 2). Providing that the time required to achieve a half of the dose was similar (arrow 1), wide variability of bout durations depended mostly on the time required to reach Δ*t*° from 5°C to 10°C. Conceivably, longer second halves of the bouts were likely due to fatigue, which typically manifests as slowing down of physical activities at the later stages of training sessions, which can result in reduction of walking speed and shorter distance of walking [29].

In order to test whether fatigue contributes to a longer duration of Δ*t*° rise from 5°C to 10°C, the participant “took” two 10°C doses consecutively using different devices to start each dose at “zero” temperature. The right panel in Figure 3 shows that the first 10°C dose was reached after 27 minutes of exercise, whereas it took about 40 minutes to take the second dose (interval 2). It also demonstrates that an extra 4 minutes (arrow 1) was required to reach 5°C at the second bout, which was likely due to fatigue accumulated during the first bout. In contrast, the durations to achieve 5°C were similar in exercise bouts taken by the recuperated participant on different days as shown (Figure 3, middle panel). Taking 2 daily MICT doses in one session also resulted in leg muscle strain reported by the participant on the next day. Therefore, this experiment corroborated that the duration required to take daily MICT dose may reflect the level of participant’s fatigue.

We further hypothesized that fluctuations of durations to “take” the same dose of MICT might reflect the diurnal variations of participant fatigability [30]. In order to test this notion the device was installed at the participant’s home to use every day in the afternoons or evenings, when it was feasible. The personalized MICT dose of 10°C was prescribed for overall 8 weeks with automatic email notifications.

Figure 4 exemplifies continuous Δ*t*° recordings from the device installed at the participant’s home. An automatic notification was sent when Δ*t*° reached 10°C as marked by arrows, which also indicates the time of the day the MICT doses were taken. During these 15 days, notifications were received 11 times, suggesting that the dose should be taken on 11 out of 15 days. It was found that training in the morning/afternoon sessions took place on days 1, 2, 5, 7, 11, 12, and 15, and in the evening sessions (after 5 pm) on days 3, 6, 8, and 9. Crosses mark the days when the device was not used (days 4, 10, 13, and 14).

**Figure 5:**
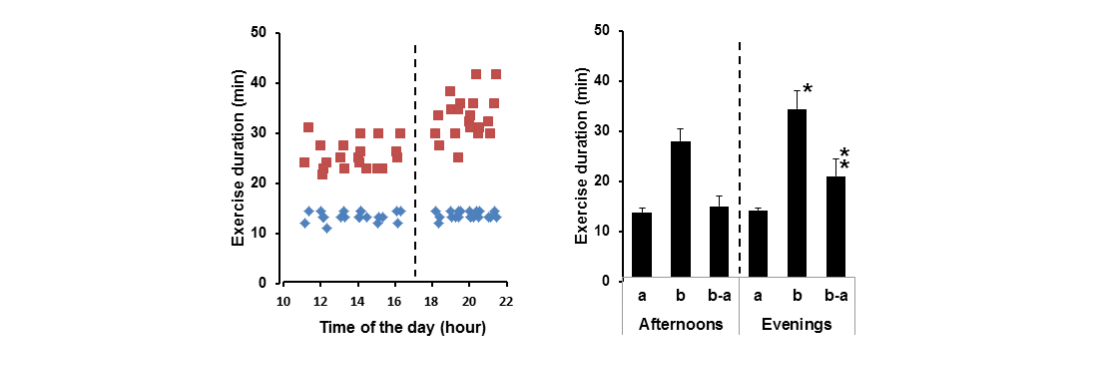
Bivariate plot of times required to achieve 5°C (diamonds) and 10°C (squares) against the times of the day, when training was performed (left). A bar chart of training times grouped into the afternoon and evening sessions (right). Bars indicate times to achieve 5°C (a) and 10°C (b), and duration of the interval between 5°C and 10°C (b-a). *P<0.05.

Over the 8 weeks of the experiment, 20 automatic notifications were received before 5 pm and 20 notifications after 5 pm. A representative bivariate plot of bout durations versus the time of day when training was performed is shown in Figure 5 (left). The plot demonstrates that Δ*t*° of 0°C to 5°C was achieved in similar periods of time regardless of the time of day, whereas the total durations of training bouts varied, particularly during the evening sessions.

The statistical analysis of training times grouped into the afternoon and evening sessions is shown in Figure 5 (right). The analysis demonstrates that in the afternoons there was no statistical difference between durations to reach Δ*t*° 0°C to 5°C and 5°C to 10°C, suggesting afternoon training as fatigue-free. In contrast, the second halves of evening training sessions, 13.5 (SD 0.5) minutes, lasted 50.9% longer than the first halves 20.3 (SD 3.5) minutes (*P*<0.05). The longer Δ*t*° 5°C to 10°C in the evenings, 14.3 (SD 2.1) minutes, compared to the afternoons, 20.3 (SD 3.5) minutes (*P*<0.05), resulted in significantly longer total durations of training of 27.4 (SD 2.4) minutes and 33.8 (SD 3.6) minutes before and after 5 pm, respectively (*P*<0.05). This data suggests the diurnal fatigability developed in the evenings results in longer durations of training required to take the same MICT dose as in the afternoons. Other participants of this study tested the system at home for 1 week. The individual Δ*t*° produced at the MICT bouts exemplified in Figure 3 (left) were set as the thresholds for automatic notifications, which were received on 4.2 (SD 1.2) days per week (N=6). Analysis of these recordings demonstrated that the mean duration of training (afternoons and evenings) was 30.3 (SD 4.1) minutes (N=25), as it was expected for these doses of training.

## Discussion

### Principal Findings

Here we demonstrated the feasibility of an objective assessment of exercise-induced fatigue and fatigability using innovative technology and protocols. Since the measurable parameters were digitalized, remote management was possible including the online monitoring of training in real-time and automated notifications by email. Together, these suggest a potential value of this approach for the remote management of CR over the Internet.

The method was based on the simultaneous monitoring of HR and LWO by Δ*t*°. While HR is a conventional physiological indicator of exercise intensity [31], it cannot be used on its own to measure the amount of training such as energy expenditure [32]. The Δ*t*° is a unique characteristic pertinent to MedExercise technology allowing for the quantification of exercise intensity and volumes [24,25]. Since a direct correlation between HR and LWO during short training bouts was shown previously [24], the parallel measurement of HR and LWO was used as a standard approach to measure the intensity and amount of exercise, respectively [26].

A main finding of this study is that fatigue can manifest as an inverse correlation between HR and LWO during a sufficiently long and intensive training and can serve as a marker of fatigue development. At steady-state HR, the reduction of Δ*t*° may indicate muscle fatigue, or inability to maintain a constant LWO level otherwise, whereas maintenance of the same LWO requires additional physical efforts from the user. The latter resulted in increasing exercise intensity, as seen by a rise in HR. Therefore, the point of time when either LWO began dropping and/or HR started increasing might be considered as an objective indicator of fatigue setting during continuous exercise training.

This study demonstrates that starting training at moderate HR may not warrant the same level of intensity and performance for the entire duration of a 30-minute bout. It suggests the need for full-duration testing at the intended intensity to ensure fatigue-free exercising and prevent negative effects of fatigue on performance, particularly in untrained participants [9]. For example, in order to ensure sustainability of MICT, the doses should be based on fatigue-free performances for the entire 30-minute duration of the exercise bout [3,4]. However, long durations might not be feasible with the current methods of fitness testing, because they are based on the measurement of HR and/or VO_2_ upon incremental (graded) increases of exercise intensity by adjusting cycle ergometer resistance, speed of treadmill belt, or pace of over ground walking [33].

In contrast to the conventional fitness testing approach where HR is a measurable variable, we measured LWO at the desirable HR, thereby removing the limitation of fitness test duration and allowing for the selection of personalized fatigue-free MICT regimens. Further, low-sensitivity recordings enabled expression of personalized daily MICT doses as single numbers. Duration of taking the same MICT dose on different days indicated relative levels of fatigability and hence could be used for the objective assessment of fatigability in health care settings. An advantage of digital dosage is the capacity for the automated notification of dose “taking” and monitoring of compliance in the network of participants remotely, which might be useful in reducing the costs of CR services [2].

### Limitations

The limitations of this study include a small sample size, a relatively short duration of intervention, and participation of healthy volunteers. Such limitations might be expected for the study focused on the development of application protocols using new CR technology. Therefore, this study should be considered as a validation study for innovative methods of measuring exercise performance and associated fatigue. Further research is warranted to establish the feasibility of this approach in larger cohorts of participants, including patients with chronic diseases and other medical conditions preventable and treatable by regular exercise training.

### Conclusion

This study showed a new approach of assessing exercise-induced fatigue by monitoring the user's work output. Since it is based on easy measurable parameters (Δ*t*° and HR), it can be applied as an additive or possibly alternative method to the subjective rating of perceived exertion and VO_2_-based fitness testing. The demonstrated feasibility of personalized exercise dosage and monitoring of compliance at distance may facilitate the delivery of exercise interventions in general and special populations.

## Abbreviations

BPM: beats per minute
CR: cardiac rehabilitation
HR: heart rate
HRmax: maximal heart rate
LWO: leg work output
MICT: moderate-intensity continuous training
RPE: perceived exertion
VO2: oxygen uptake
VO2max: maximal oxygen uptake
